# Health providers’ experiences, perceptions and readiness to provide HIV services to men who have sex with men and female sex workers in Uganda – a qualitative study

**DOI:** 10.1186/s12879-019-3713-0

**Published:** 2019-03-05

**Authors:** Joseph K. B. Matovu, Geofrey Musinguzi, Juliet Kiguli, Fred Nuwaha, Geoffrey Mujisha, Joshua Musinguzi, Jim Arinaitwe, Rhoda K. Wanyenze

**Affiliations:** 1 Department of Community Health and Behavioral Sciences, Makerere University School of Public Health, P.O. Box 7072, Kampala, Uganda; 2 Department of Disease Control and Environmental Health, Makerere University School of Public Health, Kampala, Uganda; 3 Most-at-Risk Populations (MARPs) Network, Kampala, Uganda; 4 AIDS Control Program, Ministry of Health, Kampala, Uganda

**Keywords:** Perceptions, Experiences, Health providers, MSM, FSWs, Uganda

## Abstract

**Background:**

Access to HIV services among men who have sex with men (MSM) and female sex workers (FSWs) remains suboptimal globally. While the reasons for this dismal performance have been documented, limited evidence exists on the experiences, perceptions and readiness of health providers to provide HIV services to MSM and FSWs.

**Methods:**

This analysis uses data collected from 48 key informants (health providers in public and private health facilities) as part of a larger study conducted in 12 districts of Uganda between October and December 2013. Data were collected on health providers’ experiences and readiness to provide HIV services to MSM and FSWs and their perceptions on the effect of existing legislation on HIV services provision to MSM and FSWs. Data were captured verbatim, transcribed and analyzed following a thematic framework approach.

**Results:**

All health providers reported that they had ever provided HIV services to FSWs and a majority of them were comfortable serving them. However, no health provider had ever served MSM. When asked if they would be willing to serve MSM, nearly three-quarters of the health providers indicated that they would be bound by the call of duty to serve them. However, some health providers reported that they “*would feel very uncomfortable*” handling MSM because they engage in “*a culture imported into our country*”. A majority of the health providers felt that they did not have adequate skills to effectively serve MSM and called for specific training to improve their clinical skills. There were mixed reactions as to whether existing criminal laws would affect MSM or FSWs access to HIV services but there was agreement that access to HIV services, under the existing laws, would be more constrained for MSM than FSWs since society “*does not blame FSWs [as much as it does] with MSM*”.

**Conclusion:**

A majority of the health providers were generally comfortable serving FSWs but there were strong homophobic tendencies towards MSM. A majority of the health providers lacked skills in how to handle MSM. Interventions aimed at improving health providers’ skills in handling MSM while minimizing the negative attitude towards them are urgently needed.

**Electronic supplementary material:**

The online version of this article (10.1186/s12879-019-3713-0) contains supplementary material, which is available to authorized users.

## Background

Based on the recent 2017 Global AIDS Update, the world is on track to achieve the global 90–90-90 targets by 2020. By the close of 2016, 70% of people living with HIV knew their HIV status; 77% of people living with HIV who knew their HIV status were accessing antiretroviral therapy, while 82% of people accessing treatment had suppressed viral loads [1]. Despite this level of progress, HIV infections remain much higher among some population sub-groups than in the general population, including gay and other men who have sex with men (MSM) and female sex workers (FSWs). The Joint UN Program on HIV/AIDS (UNAIDS) estimates that between 40 and 50% of new HIV infections that occurred in 2016 globally were believed to have originated from these key populations and their immediate sexual partners [1]. Besides, HIV prevalence remains much higher in these populations than in the general population, with HIV prevalence estimated to be 12–19 times higher than in the general population [2]. In Uganda, for instance, while HIV prevalence in the general population stands at 7.3% [3], HIV prevalence ranges between 33 and 37% among FSWs and between 12.2 and 13.7% among MSM [4]. In Tanzania, while the average national HIV prevalence stands at 4.7%, studies show that HIV prevalence among MSM ranges between 11.1 and 30.2% [5, 6]. Similarly, in Ghana, HIV prevalence among MSM is about 17 times higher than the national average (17% vs. 1.3%) [7]. Despite the high levels of HIV prevalence among MSM and FSWs, the coverage of appropriate HIV prevention, care and treatment programs for these key populations remains sub-optimal globally [8–11]. In some countries, only 31.2% of MSM [10] and 37.9% of FSWs [11] have been linked to HIV care, presenting a missed opportunity for harnessing the preventive benefits of antiretroviral therapy in reducing HIV transmission in these high-risk populations.

Among MSM specifically, prior studies have implicated an unwelcoming health care system as the primary reason for the low access to HIV services [12, 13]. Fear of being exposed as MSM, health workers’ homophobic tendencies, previous encounters with the health system, rumors in the gay community and legislation around homosexuality have continued to make MSM less visible in HIV care and treatment programs [13]. Indeed, a recent study among MSM and FSWs in Uganda found that 72.9% of MSM were not comfortable disclosing their sexual orientation to providers while 81.1% felt that providers did not respect MSM [14]. In the same study, more than half of the MSM reported that they experienced difficulties in accessing HIV services [14]. In Ghana, Kushwaha et al. [7] found that MSM were not well understood by the healthcare providers and that MSM did not feel that healthcare providers cared about them. In line with these findings, Kennedy et al. [15] found that perceived and experienced stigma from healthcare settings, particularly around sexual identity, led to delayed care-seeking, travel to more distant clinics and missed opportunities for appropriate HIV services among HIV-positive MSM. Evidence from previous research on this subject [16, 17] suggests that health workers have limited skills and knowledge about how to handle MSM, and that many health workers seem to be unwelcome to MSM [18]. A qualitative assessment of health seeking practices among MSM in Malawi found that health providers lacked awareness and self-efficacy to provide care in the face of limited information and political support [19]. In the same study, service providers reported concerns of adverse repercussions related to the provision of services to MSM, including being labeled as MSM themselves [19]. In addition, research from Kenya suggests that health care providers often lack professional training on specific health needs of MSM and appropriate risk reduction counseling, leaving them inadequately equipped to provide these needed services [20].

Among FSWs living with HIV in low- and middle-income countries, challenges in linking to appropriate HIV prevention, care and treatment services continue to hamper their access to HIV services, with the result being sub-optimal linkage to and uptake of HIV care services by FSWs [20]. A study conducted in Cameroon found that while coverage of antiretroviral therapy among HIV-positive individuals in the general population was as high as 56.5%, only 13.2% of HIV-positive FSWs had been linked to HIV care [9]. Results from two systematic reviews of HIV care and treatment experiences among HIV-infected FSWs in sub-Saharan Africa show that both ART initiation and current ART use remained low between 2000 and 2015 [11, 21]. Among eligible HIV-positive FSWs, ART initiation ranged from 19% in Kenya to 48% in Rwanda while current ART use ranged from 23% in Kenya to 70% in Burkina Faso [11, 21]. Several reasons have been advanced to explain this situation; including stigma, discrimination, and the fear of the consequences of seeking HIV care or of being known to practice sex work [22]. In most settings where sex work remains illegal, many FSWs may opt not to access HIV services from public health facilities due to stigma or for fear of being arrested [23, 24] or simply due to misperceptions that health workers might want to kill them [25].

Taken together, these studies suggest that MSM and FSWs still face daunting challenges in accessing health services in general and HIV services in particular. However, while previous studies have largely focused on the experiences of the clients when they try to access HIV services, few studies have explored the attitudes and practices of health providers and their readiness to serve them. This creates a missed opportunity for improving utilization of health services among MSM and FSWs since health providers’ poor attitudes and perceptions can deter them from accessing services [26]. In this study, we assessed health service provider perceptions and experiences as well as their readiness to provide HIV services to MSM and FSWs in Uganda.

The study was conducted prior to the enactment of two critical legislations in Uganda; that is, the 2014 Anti-Homosexuality Act 2014 and the HIV and AIDS Prevention and Control Act 2014. Both legislations carried clauses which, if effected, would affect access to HIV services by MSM and FSWs. For instance, the HIV and AIDS Prevention and Control Bill (at the time) allowed for acts of involuntary HIV status disclosure which would deter people, including MSM and FSWs, from accessing HIV testing services. However, all retrogressive clauses were eventually dropped before it was passed into law. On the other hand, the Anti-Homosexuality Bill (at the time) required health workers to report any MSM who accessed health services from them or face imprisonment for failing to do so; with the resultant effect that all MSM would not access such services for fear of being arrested or prosecuted. While the retrogressive clauses were dropped from the HIV and AIDS Prevention and Control Bill before it was enacted into law and the Anti-Homosexuality Act was eventually repealed, engaging in homosexuality or sex work practices remains criminal in Uganda, based on Uganda’s Penal Code Act 1950. Thus, study findings have implications for the delivery of HIV services to MSM and FSWs in Uganda.

## Methods

### Study site

This study was conducted as part of a large mixed-methods study aimed at exploring the barriers and opportunities for improving access to HIV services among MSM and FSWs in Uganda. The methods used in the large study have been described previously [13, 23]. In brief, the larger study was conducted in 12 districts of Uganda (Kampala, Mukono, Rakai, Busia, Iganga, Mbale, Soroti, Lira, Gulu, Mbarara, Hoima and Bushenyi) based on geographical representation, regional HIV prevalence and knowledge of the existence of hotspots for most-at-risk populations, including MSM and FSWs. The information on the existing hotspots for most-at-risk populations was obtained from the Most-at-Risk Populations Network, a not-for-profit organization that links populations that are highly susceptible to HIV with providers of health and legal services in Uganda (https://marps.net/).

### Study population

The study was conducted among 48 healthcare providers working with public and private health facilities in the above-mentioned districts. These informants included representatives of civil society organizations that provide HIV services to MSM and FSWs; district health personnel including the District Health Officer and the District HIV/AIDS Focal person; as well as frontline health providers (doctors, nurses) involved in the provision of HIV services in the selected districts.

### Study design

This was a cross-sectional, qualitative study that employed key informant interviews (KIIs) to collect data from purposely selected health providers.

### Participant selection and data collection

Key informants were purposively selected from health facilities that provided health services within the selected districts. Data were collected by trained Social Scientists using pretested key informant interview (KII) guides (see Additional file 1 for a copy of the KII guide). Data collectors were trained for one-week and oriented to the study procedures while improving their appreciation of the techniques needed in conducting research among key populations. The training entailed a review of the study objectives, interviewing techniques with emphasis on special issues among key populations, and detailed instruction on how to administer the interview guides. Selected community members were invited to participate in the training to enhance understanding of the target community. Some of these community members were recruited as mobilisers and thoroughly briefed about the objectives of the study and the importance of selecting appropriate participants based on the study eligibility criteria. Data were collected on the key informants’ experiences in providing HIV services to MSM and/or FSWs; their readiness to provide HIV services to them; the skillsets they possessed in terms of handling MSM and/or FSWs during service provision; whether or not they needed additional training to effectively serve MSM and/or FSWs; and their perceptions on the effect that the existing criminal laws might have on MSM and FSWs’ access to HIV services. The investigators closely supervised data collection and conducted some of the interviews. All interviews were audio-recorded with permission from the participants and transcribed verbatim within 12 h from the time of interview.

### Data analysis

Transcribed data were entered into a Microsoft Word document in preparation for analysis. Data were initially reviewed manually following three a priori themes, including: a) health providers’ experiences in and/or their readiness to serve MSM and FSWs; b) skills needed by health workers in order to provide HIV services to MSM and FSWs more effectively; and c) effect of existing criminal laws on the provision of HIV services to MSM and FSWs. The data transcripts were extensively reviewed by JKBM and GM and line-coded with guidance from the above-mentioned themes. Disagreements were resolved through discussions and constant comparison of the coded sections of the transcripts. A code-book was created to guide the subsequent stages of the analysis. Using *Atlas.ti* (version 17), we retrieved relevant quotations that pertained to each code, and those that were considered to contain “rich textual data” were selected for use in the presentation of findings. Data analysis was guided by a thematic framework approach.

### Ethical considerations

The study was approved by Makerere University School of Public Health Higher Degrees Research and Ethics Committee and cleared by the Uganda National Council for Science and Technology. Permission was also sought from the local authorities in the selected districts.

## Results

Forty eight (48) district-level key informants were interviewed for this study from 12 districts representing different HIV prevalence zones and known hotspots for MSM and FSWs. Study findings have been grouped by theme, and for each theme, we have presented supporting quotations to illustrate the main findings.

### Health providers’ experiences and/or readiness to serve FSWs and MSM

All health providers indicated that they had ever served sex workers; however, virtually no health provider reported that they had ever served MSM. Most of the health providers indicated that they were comfortable serving FSWs although a few of them expressed some level of discomfort nonetheless. We observed that the health providers’ level of comfort with sex workers was largely due to the fact that FSWs can easily open up to them about their sexual practices*:*

*“What worked well was that they [FSWs] are free and open. They freely share information about their sexual habits and most of them are ready to protect themselves. Because I remember during the moonlight tests most turned up for tests and really requested for condoms to use. They are not ashamed to ask for them*” (KII, Mbale)

Other key informants indicated that once there is rapport between the health providers and FSWs, FSWs will always be willing to “*tell you everything that you desire to hear and because of that kind of interaction, they open up and when they come to the facility they feel very welcome*” and this helps them to come for treatment without fear.

*“For the sex workers, they are so bold and as long as they have known that they are positive they will always come for their medicines and their adherence rate is far much better than for other people for as long as they have known that they are HIV positive”*(KII, Iganga)

However, a few health providers felt that FSWs do not usually open up to them about their sexual habits because they fear that the health providers will “*talk about them*” or see them as “*people who are selling themselves … people who are spreading HIV/AIDS*” (KII, Lira). In one case, a key informant in Bushenyi district indicated how she had “*personally … called one of my midwives and … told her that ‘please we need to keep confidentiality of these patients [FSWs]’. She is now comfortable with the work and is no longer talking*”. We also noted that some health providers tend to rebuke FSWs, especially those that come for antenatal services, and this tends to make them fail to open up, as one key informant from Mbale district intimated: “*At times they [FSWs] are marginalized when they come alone for antenatal care. When you ask her about the husband, she will tell you she has no husband. Then someone would say, ‘So you are the harlots, the sex workers? … You are spreading HIV/AIDS’.*”

The other reason for failing to open up is due to the fact that sex work is not legalized in Uganda and FSWs feel that if they start to talk about it; they will be arrested and prosecuted (see the Government of Uganda’s position on this issue at: https://www.mediacentre.go.ug/press-release/arrest-prostitutes). Some health workers seem to think of FSWs as “*spoilt individuals*” which creates a distance between them and their sex worker clients. For instance, in Mbale, a key informant said of FSWs: “*To me I believe that even at this health facility some of the staffs may not feel comfortable providing services to FSWs because some of them think that these are spoilt individuals so they may not attend to them … that is why there is a need to have that training that educates them that these people are like other people in the community*.”

Since many health providers indicated that they had never served any MSM in their professional life and, therefore, did not have any experience to share, we asked them to imagine what would happen if they were to serve MSM in real life. In response, nearly three-quarters of the health providers indicated that they would be comfortable serving MSM like any other patients. For instance, in Iganga, a key informant indicated that for them, service provision is not dependent on sexual orientation; so, if an MSM were to show up at a facility for HIV services, they would provide him with services in much the same way as they would do for any other patients, reiterating, “*the basis of how we offer … HIV services is not because of what you do, we even don’t ask you how you acquired HIV, but for as long as you have HIV, then you deserve to be treated …*” (KII, Iganga). This quotation raises two important aspects: first, it reflects on the readiness of health providers to provide non-discriminatory services to all patients regardless of their sexual orientation; but it also implies that health providers might have served MSM (as part of the general clientele that they served) without knowing it. If the latter observation was true, it would imply that the health providers’ submission that they have never served MSM would not be completely true given that MSM may not reveal their sexual identities to them for fear of stigmatization or being denied services. However, as discussed elsewhere in this paper, our study was not able to tease out if these observations were true.

A majority of health providers indicated that, in their capacity as health professionals, they did not have any reservations in serving MSM if they went to tem to obtain HIV services. In Soroti district, a health provider had this to say: “*… we respect diversity, we shall not castigate such people, and if they had a need we would embrace such people and help them just like any other person*” (KII, Soroti). This informant indicated that while his organization does not have any specialized clinics for MSM, they are willing and ready to serve MSM, as any other patients, and maintain the expected level of confidentiality:

*“ … if, for example, you come up with a complication which is directly attributable to homosexuality … for instance, someone comes and the complication needs a surgical intervention, we refer that person to those people whom we know can best help them. We try to maintain confidentiality which that client deserves because everybody has that right to confidentiality and autonomy to choose the type of service to be provided. I wouldn’t want to say that we have something that is specific or specialized for the MSM but we will address them with equal dignity that they deserve as human beings”*(KII, Soroti)

Another participant from Mbarara indicated that for him as a health provider, he is obliged to serve all people regardless of the way they present, their sexual practices or sexual orientation, for that matter: “*… I am a neutral person, I don’t have feelings for any kind of people regardless of who they are [wheelbarrow pusher, female sex worker or MSM] … if I know that this is the way you want me to help you, I will definitely give you the service*.” Collectively, from a professional point of view, health providers reflected on their duty to provide HIV services to MSM without discrimination.

However, when pressed further about their level of comfort as individuals in dealing with MSM, some of the health providers expressed reservations, with some of them stating that they would rather discourage men from continuing with having sex with fellow men: “*Honestly, I am a conservative person. I wouldn’t encourage men to have sex with men, so if I had a chance, I would just encourage them to leave the act. I don’t think I need to design strategies for them to continue with their act. But if they are encouraged to seek HIV, syphilis, Hepatitis testing and seeking help from a psychologist or counselor, these will be good strategies for them*” (KII, Mbarara). These perceptions show that while health providers feel the obligation to serve MSM as any other patients; in their capacity as individuals, some of them would ideally not be comfortable serving MSM.

In line with the above-mentioned observation, a key informant in Bushenyi indicated that she “*would be quite uncomfortable [providing HIV services to MSM] … I don’t see why a man should go with a man when there are women!! Women are there and besides, this is a culture imported into our country … It would be a bit uncomfortable for me to tell a man not to sleep with his fellow man when he is already used to it, I see these things on TV, the homosexuals in Kampala, but not this end*”. This participant reported that she would not feel comfortable serving MSM, because “… *it [men having sex with men] is not our habit and culture here*”. Another informant in Gulu indicated that he would be equally uncomfortable providing HIV services to MSM because “… *when I see my fellow man doing that kind of thing [having sex with another man] – I feel very low indeed and, I for one, wouldn’t encourage a man to do that kind of thing*”. These sentiments suggest that some health providers will not feel comfortable serving MSM; confirming the high homophobic tendencies towards MSM that continue to hamper access to HIV and other health services in many health facilities in Uganda.

### Skills needed by health providers in order to effectively serve MSM and FSWs

Evidence from Kenya suggests that if health providers are trained in how to handle MSM, this can improve their knowledge about MSM needs and reduce their homophobic tendencies towards them [27, 28]. In following up on these observations, we asked health providers if they had ever received any form of training on how to handle MSM or FSWs; and if not, whether they would be willing to receive such training. In response, most health providers indicated that they did not have any specific skills on how to handle MSM or FSWs in clinical settings, and tended to handle them like any other clients. Some health providers called for a need to be trained in how to communicate with MSM, reasoning that this could help them to serve MSM better: “*I think we are missing a lot because … there could be some kind of communication that can be used to communicate with these people that we could be missing. Thus, I don’t think we have the knowledge to help us identify these populations [or create an environment that makes them feel free to come to us] or know what to do when they come to us*” (KII, Gulu).

When asked about whether or not health providers would welcome to be trained in how to handle MSM, an informant from Iganga said such training would be acceptable and would help to “*change attitudes of some of our health workers*”. The ‘change in attitude’ referred to in the quotation would manifest in health providers’ willingness and openness to serve MSM in a non-discriminatory manner. In Mbarara, another informant suggested a need for revising the curriculum for medical and nursing students to include an emphasis on how to “*handle special groups*” such as FSWs and MSM:

*“ … some of the things I think that need to be improved in the curriculum is HIV medicine. Although HIV medicine is there, what is still missing is how to handle special groups, I am not so sure if it exists in the curriculum. Because for me I get involved in teaching undergraduates but the HIV topics I teach are the basic ones; how to serve anybody who has come for HIV services. So I think as we teach HIV medicine, consideration should also be given on how to serve these most-at-risk populations guided by research and evidence on what they need and the best way we can approach them”*(KII, Mbarara)

A few participants called for a need to be trained on gender identification among MSM, reasoning that this is because MSM adopt “*a different gender role depending on the day and circumstances*”:

*“I think that the medical practitioners should be taught issues relating to gender identification amongst MSM; because some people feel that they are of a different gender depending on the day and circumstances”*(Key informant, Kampala)

The call for ‘gender identification’ could be because of a false perception among some health providers that service delivery to MSM would have to be differentiated depending on the role played by men in the relationship or on their sexual identities, which is not true. There is no need for MSM to reveal their sexual identities prior to being served; nor are they expected to indicate what role they play in the relationship. The presence of these sentiments reaffirms the need for health providers to receive gender sensitivity training that should help to address any existing stereotypes around MSM and therefore be able to serve MSM in a non-discriminatory and non-stigmatizing manner.

However, while most of the participants indicated that they would welcome any opportunity to be trained in how to handle FSWs and/or MSM, some of them did not feel the need to be trained in how to handle MSMs: “*I feel I am missing that training of handling FSWs who are many in our country. With homosexuals [MSM], I don’t think I would be willing to go for that training*” (KII, Bushenyi). This lack of interest in receiving training on how to handle MSM was strongly entrenched in the health providers’ cultural beliefs: “*I wouldn’t like [to imagine a] scenario where men are sleeping with men, women are sleeping with women … and some of them taking these drugs like marijuana, you really feel they could maybe benefit from the psychiatric nurses and doctors*” (KII, Kampala). Thus, while some participants did not have any objection to being trained in how to handle FSWs, some of them had strong homophobic tendencies towards being trained to serve MSM. Indeed, based on the quotation above, one can infer that these participants equated homosexuality to a mental problem that required the intervention of ‘psychiatric nurses and doctors’.

### Effect of existing criminal laws on the provision of HIV services to MSM and FSWs

As noted earlier, this study was implemented before the enactment of the Anti-Homosexuality Act 2014 and the HIV and AIDS Prevention and Control Act 2014. We asked health providers whether or not they thought such Bills, if passed into law, would affect the way MSM and FSWs access HIV and other health services. In response, some health providers thought that if such Bills were passed into law, they would definitely affect the way MSM and FSWs access services: “*Yes. It will affect [them] in some way because they [MSM or FSWs] have to keep it a secret for fear that if they open to you, you may take them to the law makers. So they will keep there and keep spreading the virus and other STIs*” (KII, Kampala). However, some other health providers did not think that the existence of these Bills – or even if they were passed into law – would affect uptake of HIV or other health services by MSM or FSWs, insisting that the health services are “*open to anybody*”, and that access to and utilization of health services does not require one to disclose their sexual practices or orientation:

*The law may hinder but I don’t think it’s the biggest factor. If you love yourself you go for the services, you don’t have to tell your neighbor that you’re a sex worker or MSM. The only implication the law has is that it keeps them in hiding but if you love yourself then you seek the service. I think services are open to anybody … these populations just need to be talked to and be informed where they can find these services and also be helped out of their stigma, otherwise there’s no discrimination between the populations we serve*(KII, Mbarara)

Indeed, when asked if the existence of any FSW- or MSM-specific legislation would affect the way they (health providers) provide health services to MSM or FSWs, majority of the health providers did not think that such legislation would affect them in any way. A key informant in Busia had this to say: “*We as service providers … it [the law] will not affect us because we have to treat all people who are sick. But I think that it has scared off the MSM from disclosing when they need services*”. Although health providers did not seem to agree on the effect of the existence of the two Bills on access to and uptake of HIV services by MSM and FSWs, there was agreement that the Bills, in their state at the time – or when passed into law – would affect MSM more than FSWs since society “*does not blame FSWs [as much as it does] with MSM*”. The societal ‘blame’ points to the cultural beliefs around anal sex and other forms of sexual behaviors practiced by MSM. Thus, while being a FSW is frowned upon in society (e.g. women who engage in sex work may be considered to be ‘spoilt’), there is less of stigma around sex work than it is with engaging in anal sex in the Ugandan society. There is a feeling that anal sex and other MSM sexual practices are ‘foreign’ and therefore not part of the Ugandan culture.

## Discussion

Our study of the health providers’ perceptions and readiness to serve MSM and FSWs in Uganda revealed four interesting scenarios: a) all health providers reported that they had ever served FSWs and a majority of them said they were comfortable serving them; b) No health provider reported that they had ever served MSM but when asked if they would be comfortable serving them (if MSM went to them for HIV services), some health providers expressed strong homophobic tendencies towards them; c) most health providers lacked skills in serving MSM and FSWs; and d) there were mixed reactions as to whether any existing criminal laws would affect MSM or FSWs’ access to and eventual utilization of HIV services.

Our finding that most health providers had favorable attitudes towards serving FSWs may not be surprising given that engaging in sex work, while illegal in Uganda, is not necessarily viewed as ‘foreign’. Indeed, many health providers found it convenient to serve FSWs since they easily open up to them about their health challenges. However, although no health provider reported that they had ever served MSM, in theory, some health providers expressed strong homophobic tendencies towards them. The expression of these tendencies can be attributed to the perception that anal sex is not part of the Ugandan culture. Homophobia – the dislike of or prejudice against homosexual people – has been identified as a barrier to HIV prevention service access among MSM [4, 29]. However, unlike in prior studies where these prejudices were felt by the clients themselves [30, 31], in our study, homophobia was expressed by the health providers which presents serious implications for the provision of HIV and other health services to MSM in Uganda [13, 23]. In a study conducted among Kenyan FSWs, Nyblade et al. [23] found that FSWs who anticipated health worker mistreatment had significantly higher odds of avoiding non-HIV services compared to those who did not. In a review of literature conducted by Ippoliti et al. [32], FSWs who desired a pregnancy faced additional stigma from health providers who believed them to be unfit parents. In Ghana, Kushwaha et al. [7] found that MSM felt that they were not understood by the healthcare providers and that healthcare providers did not care about them. Collectively, these findings suggest a need to improve health provider and key populations’ interactions, including a change of attitude on the side of health providers, in order to enhance HIV service delivery to MSM and sex workers.

All health providers reported that they had never served MSM although it is likely that they could have served them as part of their general clientele without knowing it. This is also likely to be the case given that in selecting the study sites, consideration was made of hotspots where MSM and FSWs would most likely seek HIV and other health services. Alternatively, given the strong homophobic tendencies expressed by some of the health providers, MSM might have opted not to reveal their sexual orientation or identities to them for fear of being denied services or stigmatized. Also, since the study was conducted at a time when there were two pending legislations that required health providers to report any MSM that they served, there is a possibility that some health providers might have opted to deny that they had ever served MSM for fear of being apprehended. The Anti-Homosexuality Bill, for instance, stipulated stiffer punishments for health providers who came in contact with MSM (when they accessed services from them) but failed to report them to higher authorities. It is, important to note, however, that our study was not able to tease out whether or not these observations were true; calling for further research to fully document the experiences of health providers who have actually ever served MSM in order to capture their real life experiences as opposed to those based on hypothetical imaginations.

In our study, most health providers accepted that they did not have the requisite skills necessary to provide HIV services to MSM and FSWs (but most especially skills to handle MSM) and most of them felt that they needed to be trained in how to handle MSM, including how to effectively communicate with them. As has been documented elsewhere, lack of cultural and clinical competent by health providers can affect MSM and FSWs utilization of HIV and other health services [19, 33]. Although some of the health providers did not feel the need to be trained in how to handle MSM issues, evidence from prior studies suggest that health providers who have received sensitivity training [27, 28, 34] express greater acknowledgement of MSM patients in their clinics, endorse the need to treat MSM patients with high professional standards and demonstrate sophisticated awareness of the social and behavioral risks for HIV among MSM than those who have never attended such trainings. These findings underscore the need to target all health providers with trainings in order to improve their knowledge about MSM health needs and reduce homophobic attitudes towards them.

We found mixed reactions as to whether the existing legal framework on the provision of health services to MSM or FSWs would affect their access to HIV or other health services. While some health providers felt that the existing criminal laws would impede access to and utilization of HIV services by FSWs – and most importantly, MSM – others felt that the existing legal framework would not service access or uptake since the provision of such services is not dependent on one’s sexual practices or their sexual orientation. However, there was agreement – at least among some health providers – that any existing legislation would affect access to HIV services by MSM more than it would do for FSWs since society “does not blame [FSWs] as much as it does with MSM”. These findings were in agreement with findings reported by Sekoni et al. [35] who found that the enactment of the Same-Sex Marriage Prohibition Act in Nigeria in 2014 influenced the way doctors and other health professionals provided health services to MSM. Indeed, Sekoni et al. [35] found that up to 24% of medical students agreed with the statement that health providers should not provide services to MSM, and 18.2% agreed that MSM should not have access to HIV prevention services. Similar findings have been reported by Schwartz et al. [36] who found that the proportion of MSM who feared to seek health care from formal healthcare settings increased from 25% before to 38% after the Same-Sex Marriage Prohibition Act was enacted in Nigeria in 2014. Thus, although some of the health providers in our study did not think that the existing criminal laws would affect MSM or FSWs access to and utilization of HIV and other health services, there is evidence to show that the existence of such legislation can reduce access to health services [29]. These findings call for a need to sensitize health providers about the inherent rights of MSM – and FSWs alike – and the need to provide HIV and other health services to them without discrimination.

Our findings have public health and policy implications. From a public health point of view, our findings call for a need to improve health providers’ ability to serve MSM and FSWs without prejudice, particularly through sensitivity training. Evidence from sensitivity trainings conducted in Kenya [27, 28] attests to the fact that such trainings can improve the way health providers provide services to these key populations. Thus, programs targeting MSM and FSWs should incorporate sensitivity trainings to improve access to and utilization of health services by MSM and FSWs. From a policy perspective, our findings are in direct consonance with findings reported by Duvall et al. [37] which call for the enactment of policies that create a favorable environment within which MSW and FSWs can freely access services without fear of being prosecuted. Such policies should also help to address stigma and discrimination that continue to hamper effective access to and eventual utilization of health services by MSM and FSWs.

Our study had a number of limitations and strengths. In terms of limitations, the fact that this is a qualitative study that explored perceptions and experiences of serving MSM and FSWs from purposely selected health providers may limit generalizability of the study findings. We tried to improve external validity by interviewing health providers from both public and private health facilities across 12 districts; and we believe that the study findings may, in part, represent the perceptions of most Ugandan health workers when it comes to serving or readiness to serve MSM and FSWs. The other limitation is that we did not interview any health providers who had ever served MSM, meaning that the perceptions about MSM, as expressed in this study, were largely hypothetical. However, it is likely that some health providers could have ever served MSM inadvertently since disclosure of one’s sexual orientation or identity is not a requirement for service access; or some health providers could have ever served MSM but did not want to acknowledge this due to fear of being associated with MSM in a society that treats anal sex and other sexual behaviors of MSM as ‘foreign’ [27]. Nevertheless, our study was not able to confirm if these aspects were true. Future studies should include interviews with health providers who *actually* admit that they have ever served MSM so as to capture their experiences.

Despite these limitations, we believe that our study provides valuable findings from the perspective of health providers. This is because earlier studies concentrated more on the experiences and perceptions of MSM or FSWs in seeking health care, and, although some of the sentiments expressed in this paper were also captured in those studies, the findings were not corroborated with interviews conducted among health providers. Thus, our study presents some form of triangulation of data on the experiences already shared by clients and, to some extent, confirm some of the fears already expressed by the clients. The other strength about our study is that interviews were conducted in 12 districts with differing HIV prevalence levels; representing a wide spectrum of views expressed by the health providers in as far as serving or readiness to serve both MSM and FSWs is concerned.

## Conclusion

Our study shows that a majority of the health providers were generally comfortable serving FSWs but there were strong homophobic tendencies towards MSM. A majority of the health workers lacked skills in how to handle MSM and called for a need to be trained in how to effectively serve MSM and FSWs. Interventions aimed at improving health providers’ skills in handling MSM while minimizing the negative attitude towards them are urgently needed.

## Additional file

Additional file 1: Key Informant Interview Guide. This KII guide includes questions that were administered to health providers as part of a large mixed-methods study aimed at exploring the barriers and opportunities for improving access to HIV services among MSM and FSWs in Uganda. Only questions that explored health providers’ experiences, perceptions and readiness to provide HIV services to MSM and FSWs were analyzed for this paper. (PDF 193 kb)

## Abbreviations

AIDS: Acquired Immune Deficiency Syndrome
ART: Antiretroviral therapy
FSW: Female Sex worker
HIV: Human Immunodeficiency Virus
KII: Key Informant Interview
MSM: Men who have sex with men
STI: Sexually transmitted infection
