# Supplementary material for: Health providers’ experiences, perceptions and readiness to provide HIV services to men who have sex with men and female sex workers in Uganda – a qualitative study
Source: BMC Infect Dis. 2019 Mar 5;19:214. doi: 10.1186/s12879-019-3713-0 (PMC6400025; doi:10.1186/s12879-019-3713-0)
Supplement: Supplementary file 1 — Key Informant Interview Guide. This KII guide includes questions that were administered to health providers as part of a large mixed-methods study aimed at exploring the barriers and opportunities for improving access to HIV services among MSM and FSWs in Uganda. Only questions that explored health providers’ experiences, perceptions and readiness to provide HIV services to MSM and FSWs were analyzed for this paper. (PDF 193 kb) [file 12879_2019_3713_MOESM1_ESM.pdf]

BARRIERS AND OPPORTUNITIES FOR INCREASING ACCESS TO HIV SERVICES AMONG SELECTED MOST AT RISK POPULATIONS IN UGANDA

Key Informant interview guide

1. If you had to rate your comfort level administering HIV prevention services to people who visit your facility on a scale from 1 to 5 (5 being most comfortable), how would you rate yourself?

1a. Are there certain risk groups that you feel more comfortable working with than others?

1b. What is it about working in HIV prevention that makes you feel most or least comfortable?

1c. What aspects of your previous or ongoing training/education could be improved to enhance your comfort level administering HIV prevention services? What professional skills would you like to strengthen to improve your service provision?

2. If you had to rate your comfort level administering HIV prevention services to (1) men who have sex with men and to (2)Sex Workers on a scale from 1 to 5 (5 being most comfortable), how would you rate yourself?

2a. When you are working with MSM/SW, do you feel comfortable asking about their sexual habits and partners? In general, do they feel comfortable volunteering this information to you on their own?

2b. What is it about working with MSM/SW that makes you feel most or least comfortable?

2c. When making referrals for medical and mental health follow-up services, how well do your MSM clients comply when compared to other high-risk groups (in other words, is it easy or difficult to get MSM/SW to come back for follow-up care)?

3. How well do you feel you've been trained on working with men who have sex with men and sex workers in HIV clinical settings?

3a. Is there training or a set of skills that you feel like you're missing when it comes to providing services for these population?

4. How would you describe the experiences of MSM and SW with health care facilities and resources in this area?

4a. Can you name specific centers that are especially welcoming to MSM/SW? Can you name some facilities that have a reputation for not being welcoming to MSM/SW?

4b. What type of health care facilities make MSM/SW feel safe?

4c. What actions and behaviors do employees at local health care agencies do to make MSM feel unwelcome?

5. As HIV service providers, how much concern do you think your community has for the health outcomes of men who have sex with men/SW?

5a. When compared to other risk groups like heterosexual women, heterosexual men, infants born to infected mothers, how much emphasis do you think is placed on prevention among MSM/SW?

6. What are some of the ways that you encourage MSM/SW to go about protecting themselves from HIV infection?

6a. Do you encourage them to get tested for HIV regularly?

6b. Do you encourage them to use PrEP or PeP?

6c. Do you encourage them to use lubricant or wear condoms with each of their sex partners?

6d. Do you encourage them to learn the status of their sex partners?

7. We would like to explore your interest in a series of possible packages of care for your gay and bisexual male patients. Which of the following services in a package of care do you think your patients would be most responsive to?
   a. Couples voluntary counseling and testing; condoms and lubricant; health information on mobile phones
   b. PrEP; condoms and lubricant; health information on mobile phones
   c. Circumcision; condoms and lubricant; health information on mobile phones
   d. LGBT sensitization training for medical providers; condoms and lubricant; health information on mobile phones
   e. Some combination of the above package elements

8. What are some things that get in the way of MSM/SW protecting themselves from HIV infection?

8a. Does religion ever present a problem for MSM protecting themselves from HIV infection?

8b. Does culture or ethnic background ever gets in the way of HIV prevention strategies?

8c. What about family or community stigma around the idea of homosexual behavior? Does that ever deter them from seeking resources that could help them prevent HIV infection?

9. Are there formal or informal groups of men who have sex with men that provide HIV prevention support?

9a. In what ways do they help MSM/SW protect themselves from HIV infection?

9b. In what ways to they make it difficult for MSM/SW to protect themselves from HIV infection?
